# The Association of rs1898830 in *Toll-Like Receptor 2* with Lipids and Blood Pressure

**DOI:** 10.3390/jcdd7030024

**Published:** 2020-07-08

**Authors:** Pia Chedid, Ali Salami, Said El Shamieh

**Affiliations:** 1 Department of Medical Laboratory Sciences, Faculty of Health Sciences, University of Balamand, Beirut 1100-2807, Lebanon; pia.chedid@fty.balamand.edu.lb; 2 Rammal Hassan Rammal Research Laboratory, Physio-toxicity (PhyTox) Research Group, Lebanese University, Faculty of Sciences (V), Nabatieh 6573/14, Lebanon; a.salami@ul.edu.lb; 3 Department of Medical Laboratory Technology, Faculty of Health Sciences, Beirut Arab University, Beirut 115020, Lebanon

**Keywords:** lipids, hypertension, cardiovascular diseases, toll-like receptor 2, rs1898830, single nucleotide polymorphisms, association analysis

## Abstract

Background and Objective: Toll-like receptors (TLRs) are important components of the innate immune system, involved in establishing immunity to infections. Apart from being implicated in immunity, numerous studies have reported that many TLRs, including TLR2, are involved in the pathogenesis of cardiovascular diseases and their risk factors. Since rs1898830 is associated with TLR2-mediated cellular activation, we aimed to study its association with CVD risk factors, such as lipid levels and hypertension. Methods: A cross-sectional study was conducted on 460 individuals free from chronic diseases. Clinical and biological data were collected and DNA was extracted and genotyped using Kompetitive allele specific PCR (KASP™). Multiple logistic regression models, adjusted for six covariates, were used. A power calculation analysis was also performed. Results: We found that rs1898830 in *TLR2* was positively associated with hypertension (OR = 2.18, *p* = 0.03) and negatively associated with high-density lipoprotein cholesterol (OR = 0.66, *p* = 0.05). In contrast, no relation was found with total cholesterol and low-density lipoprotein cholesterol. Conclusion: The present results provide additional evidence supporting the implication of TLR2 in CVD risk factors.

## 1. Introduction

Cardiovascular diseases (CVDs) are the leading worldwide cause of death [1]. In 2016, CVDs affected around 121.5 million individuals [1]. Smoking, lack of physical activity, hypertension (HTN), obesity, dyslipidemias and type 2 diabetes are modifiable risks factors for CVDs, while age, gender and family history are non-modifiable factors [2,3]. Regarding family history, several studies have shown the importance of common polymorphisms in modulating the predisposition to CVDs [4]. The study of candidate genes in lipid metabolism [5], such as *APOE*, has shown that in subjects carrying the *APOE4* isoform, plasma cholesterol, low-density lipoproteins and apolipoprotein B (apoB) were the highest, as compared to individuals with the *APOE3* or *APOE2* isoforms [6]. The study of other genes, such as genes of fibrinolytic proteins [7], angiotensin-converting enzyme [8] and a cluster of differentiation 14 (CD14) [9] has also shown promising results.

Inflammatory mechanisms are triggered early during the onset of CVDs, especially atherosclerosis-related CVDs [10]. Toll-like receptors (TLRs) recognize pathogen-associated molecular patterns (PAMPs) and are expressed by myeloid cells, such as monocytes and macrophages, as well as endothelial cells [11]. They trigger immediate, as well as long-term, immune defense for the host [12]. In ApoE(+/−) mice, for example, the genetic deficiency in TLR2 reduced diet-and/or pathogen-associated atherosclerosis [13]. Interestingly, we have previously shown that the G allele of single nucleotide polymorphism (SNP) rs2569190 in *CD14*, a cofactor for Toll-like receptor 2 (TLR2), is associated with increased levels of total cholesterol and low-density lipoprotein cholesterol (LDL-C), and decreased levels of high-density lipoprotein cholesterol (HDL-C). 

Numerous studies have reported that many TLRs, including TLR2, are involved in the pathogenesis of CVDs, including atherosclerosis [14,15,16,17,18,19]. Specifically, the expression of TLR2 is significantly increased in endothelial cells from atheroma plaques [20]. Since rs1898830 is associated with TLR2-mediated cellular activation [21], we hypothesized that this single nucleotide polymorphism could also be associated with increased risk of CVDs risk factors. Thus, in the current study, the association of rs1898830 in *TLR2* with lipid traits and hypertension (HT) was investigated in 460 individuals from the general Lebanese population.

## 2. Material and Methods

### 2.1. Study Population

The recruitment procedure and the genetic protocols were approved by the Institutional Review Board of the Beirut Arab University (2019-H-0091-HS-R-0360). All participants gave their written informed consent. Our participants were unrelated individuals free of chronic disease (cardiovascular or cancer) with recruitment taking place from 2015 to 2016 in a major tertiary care hospital. 

### 2.2. Clinical and Biological Data Collection

All the measurements, as shown in Table 1; Table 2, were performed as described previously [9,22]. Nuclear DNA from whole-blood samples was extracted using the QIAamp DNA blood mini kit (Qiagen, Hilden, Germany) and genotyped for rs1898830 in TLR2 using Kompetitive allele specific PCR (KASP™). The quality of DNA was assessed through electrophoresis on 1% agarose gel. In all genotyping experiments, positive and negative controls were also added. Values above 115 mg/dL, ≥190 mg/dL and ≥150 mg/dL for LDL-C, total cholesterol (TC) and triglyceride (TG), respectively, were considered abnormally high. Values below 50 mg/dL and 40 mg/dL for HDL-C levels in females and males, respectively, were considered abnormally low. A systolic blood pressure of 130 mmHg and/or diastolic 85 mmHg was considered normal HTN.

### 2.3. Statistical Analyses

The statistical analyses were performed using the SPSS (IBM Corp., Released 2013, SPSS Statistics for Windows Version 22.0, Armonk, NY, USA), whereas continuous variables were presented as mean ± SD and categorical variables were shown as numbers followed by percentages. To determine if the genotypes of rs1898830 in *TLR2* were in Hardy–Weinberg equilibrium (HWE), a chi-squared goodness-of-fit test was performed.

A multivariate logistic regression model was used to study the association between rs1898830 in TLR2 and lipid traits and HTN. The model was corrected for six covariates (physical activity, marital status, body mass index, smoking, age, and gender) under the assumption of an additive model (AA vs. GA vs. GG). The sample size needed GPower 3.1.9.4 software [23] and the calculations showed that 409 individuals were needed to reach a statistical power of at least 0.90 in a two-sided test with α = 0.05 and an effect size of 0.2.

## 3. Results

### 3.1. Characteristics of the Studied Participants

All measured criteria are listed in Table 1 and Table 2. The total number of the individuals was 460, divided into two groups: 292 females and 168 males, making a ratio female to male of 1.7:1. Concerning demographic data, the average age of the participants was 40.6 years old. The percentage of married participants and of smokers was, respectively, around 70% and 25%, and very few were exercising once per week, as shown in Table 1.

Concerning clinical and genetic criteria, around 50% of the participants had high TC, high LDL-C levels, and low HDL-C, as shown in Table 2. The minor allele frequency rs1898830 was 0.34 with the AA genotype being the most prevalent, followed by AG and GG, as shown in Table 2. Moreover, the allelic frequencies were in line with HWE (*p* = 0.07).

### 3.2. Association of rs1898830 in TLR2 with Lipids and Hypertension

The G allele of rs1898830 in *TLR2* was associated with decreased HDL-C levels (OR = 0.66 and *p* = 0.05, Table 3) and an increased risk of HTN (OR = 2.18 and *p* = 0.03), as shown in Table 3. Individuals with the GG genotype have 33% lower HDL-C levels when compared with individuals with AA and AG. Similarly, individuals with the GG genotypes were at twice the risk of developing HTN. In addition to rs1898830, whereas smoking status was also associated with decreased HDL-C levels (OR = 0.39, *p* < 0.001), women were at a higher risk of developing HTN than men (OR = 1.96, *p* = 0.003). In contrast to age, gender and marital status, which were not associated with HDL-C, body mass index and smoking showed significant relations, as shown in Table 2. No significant association was seen for rs1898830 in *TLR2* with TG (*p* > 0.05, data not shown).

## 4. Discussion

In the current study, the G allele of rs1898830 in *TLR2* was found to be associated with decreased levels of HDL-C and increased risk of HTN. Individuals with the GG genotype had 33% lower HDL-C levels when compared with individuals with AA and AG. Similarly, individuals with the GG genotypes were at twice the risk of developing HTN.

Human *TLR2* is located on the long arm of chromosome 4 (4q31.3). TLR2 is a cell surface receptor responsible for the recognition and binding of a variety of microbial components, such as lipoteichoic acid and bacterial lipoproteins from Gram-positive bacteria [24]. The expression of TLRs, especially TLR1, TLR2 and TLR4, is significantly increased in endothelial cells from atheroma plaques [20]. In peripheral monocytes of nondiabetic hypertensive patients, the control of HTN significantly decreases the expression of TLR2 and TLR4 [25]. The contribution of TLR2 and TLR4 to hypertensive heart failure has also been studied in a mouse model of sustained pressure overload. *TLR2*−/− mice were protected against cardiac hypertrophy, fibrosis and dysfunction induced by sustained pressure overload, as compared to wild type mice [16].

It is well documented that HDL-C prevents the progression of atherosclerosis by several mechanisms, such as reducing the inflammatory reaction, removing cholesterol from foam cells and inhibiting LDLs oxidation [26]. On the other hand, HTN might participate in the progression of atherosclerosis by causing endothelial injury via prolonged extra pressure on the arterial walls. Arterial damage is considered an early step leading to atheroma plaque build-up and blood vessel narrowing during atherosclerosis [27]. Thus, atherogenesis might be triggered at early stages by the association of rs1898830 with decreased HDL-C and increased risk of HTN. Several studies have implicated certain TLRs polymorphisms with inflammatory diseases [28,29,30]. The SNP rs1898830, known as -15,607A/G, is in the first intron of the *TLR2* gene. In chronic obstructive pulmonary disease (COPD), rs1898830 was associated with an increase in the number of inflammatory cells in the sputum of the COPD patients and with a decrease in lung function [31]. However, the exact mechanism of the *TLR2* rs1898830 SNP remains unclear. rs1898830 has been associated with TLR2-mediated cellular activation [21]. Therefore, one hypothesis could be that the rs1898830, known as -15607A/G might alter the expression or functionality of TLR-2, leading to an increase in macrophages and endothelial cell number and/or function, thus enhancing the innate immune response that participates in the pathophysiology of atherosclerosis. Further studies are needed to better understand the mechanism underlying the association of rs1898830 in *TLR2* with CVDs.

The findings of the present study are strengthened by the fact that the regression models used in this study were adjusted for several confounding independent variables. However, the lack of replication in larger independent populations is the main limitation of our results.

The current study is one of many reporting a link between components of the innate immune system and CVDs [32,33]. It is known that activated innate immune system elements and dysfunction in metabolic pathways could lead to the chronic inflammation, resulting in CVDs [32,33].

## 5. Conclusions

In conclusion, the results herein indicate that rs1898830 in TLR2 is negatively associated with HDL-C and positively correlated with HTN. This link might highlight that rs1898830 could be implicated in the pathogenesis of CVDs.

## Figures and Tables

**Table 1:** Demographic characteristics of the study participants.

Characteristics	Participants (*n* = 460)
Age	40.60 ± 14.16
Gender *n* (%)	
Male	168 (36.5)
Female	292 (63.5)
Smoking status *n* (%)	
Non-smoker	332 (72.2)
Past smoker	6 (1.3)
Current smoker	122 (26.5)
Marital Status *n* (%)	
Single	121 (26.3)
Married	321 (69.8)
Divorced	18 (3.9)
Physical Activity *n* (%)	
<1 per week	345 (75.0)
1 per week	52 (11.3)
≥2 per week	63 (13.7)

Values are arithmetic mean ± SD for continuous variables. Categorical variables are shown as numbers (*n*) and percentages (%). *n*: sample size.

**Table 2:** Clinical and genetic characteristics of the study participants.

Characteristics	Participants (*n* = 460)
BMI (Kg/m^2^)	25.71 ± 4.98
Total cholesterol (mg/dl)	181.41 ± 40.94
High total cholesterol levels *n* (%)	241 (52.4)
LDL-C (mg/dl)	117.39 ± 33.52
High LDL-C levels *n* (%)	238 (51.7)
HDL-C (mg/dl)	45.53 ± 14.61
Low HDL-C levels *n* (%)	270 (58.7)
Triglycerides (mg/dl)	145.96 ± 124.34
High triglycerides levels *n* (%)	174 (37.8)
SBP (mmHg)	132.07 ± 15.89
DBP (mmHg)	67.82 ± 9.12
Hypertension *n* (%)	255 (55.4)
MAF	
MAF of rs1898830 in *TLR2*	0.34
AA *n* (%)	197 (42.8)
GA *n* (%)	212 (46.1)
GG *n* (%)	51 (11.1)

Values are arithmetic mean ± SD for continuous variables. Categorical variables are shown as number (*n*) and percentages (%). *n*: sample size. BMI: body mass index, LDL-C: low-density lipoprotein cholesterol, HDL-C: high-density lipoprotein cholesterol, SBP: systolic blood pressure, DBP: diastolic blood pressure, MAF: minor allele frequency, TLR2: Toll-like receptor 2.

**Table 3:** Multiple logistic regression analysis of rs1898830 in *TLR2* with hyperlipidemia and hypertension.

Variables	Total Cholesterol		LDL Cholesterol		HDL-Cholesterol		Hypertension	
	OR (95% C.I.)	*p*	OR (95% C.I.)	*p*	OR (95% C.I.)	*p*	OR (95% C.I.)	*p*
**rs1898830AA**	1		1		1		1	
GA	1.03 (0.63–1.67)	0.914	1.29 (0.79–2.09)	0.300	0.66 (0.44–1.01)	0.050	0.87 (0.58–1.32)	0.517
GG	1.40 (0.63–3.14)	0.409	1.49 (0.68–3.25)	0.319	0.74 (0.39–1.42)	0.366	2.18 (1.08–4.39)	0.030
**Age**								
<40	1		1		1		1	
≥40	0.44 (0.26–0.74)	0.002	0.39 (0.24–0.67)	0.001	0.67 (0.44–1.03)	0.071	1.12 (0.73–1.72)	0.602
**Gender**								
Male	1		1		1		1	
Female	0.74 (0.44–1.24)	0.253	1.03 (0.61–1.73)	0.915	0.79 (0.51–1.22)	0.287	1.96 (1.26–3.05)	0.003
**BMI**								
<25	1		1		1		1	
25–29.9	0.64 (0.37–1.13)	0.123	0.50 (0.29–0.88)	0.016	2.24 (1.33–3.75)	0.002	0.88 (0.54–1.45)	0.615
≥30	1.27 (0.65–2.49)	0.484	1.29 (0.66–2.55)	0.454	0.85 (0.49–1.44)	0.542	1.45 (0.83–2.51)	0.192
**Marital status**								
Single	1		1		1		1	
Married	0.64 (0.35–1.16)	0.138	0.89 (0.49–1.56)	0.679	0.66 (0.40–1.10)	0.112	0.65 (0.39–1.07)	0.090
Divorced	0.85 (0.27–2.72)	0.785	1.28 (0.39–4.14)	0.677	2.56 (0.75–8.73)	0.132	0.34 (0.11–0.98)	0.047
**Smoking status**								
Non-smoker	1		1		1		1	
Past smoker	0.86 (0.14–5.29)	0.866	0.89 (0.14–5.78)	0.909	0.47 (0.09–2.63)	0.391	0.23 (0.04–1.42)	0.12
Current smoker	1.83 (0.99–3.35)	0.050	1.78 (0.98–3.22)	0.057	0.39 (0.24–0.63)	<0.001	0.67 (0.42–1.07)	0.095
**Physical activity**								
<1 per week	1		1		1		1	
1 per week	0.24 (0.12–0.49)	<0.001	0.23 (0.12–0.47)	<0.001	0.42 (0.21–0.83)	0.01	0.91 (0.47–1.79)	0.788
≥2 per week	0.77 (0.39–1.52)	0.446	1.45 (0.69–3.02)	0.327	0.52 (0.28–0.97)	0.051	0.87 (0.47–1.59)	0.642

OR: odds ratio, C.I.: confidence interval, BMI: body mass index, HDL-C: high-density lipoprotein cholesterol, LDL-C: low-density lipoprotein cholesterol.
